# Influence of the Nitrogen Fertilization on the Yield, Biometric Characteristics and Chemical Composition of *Stevia rebaudiana* Bertoni Grown in Poland

**DOI:** 10.3390/molecules29081865

**Published:** 2024-04-19

**Authors:** Joanna Śniegowska, Anita Biesiada, Alan Gasiński

**Affiliations:** 1 Department of Horticulture, Wrocław University of Environmental and Life Sciences, 50-375 Wrocław, Poland; anita.biesiada@upwr.edu.pl; 2 Department of Fermentation and Cereals Technology, Wrocław University of Environmental and Life Sciences, 50-375 Wrocław, Poland; alan.gasinski@upwr.edu.pl

**Keywords:** stevia, stevioside, rebaudoside A, urea, ammonium nitrate, ammonium sulfate

## Abstract

*Stevia rebaudiana* Bertoni is a plant native to South America that has gathered much interest in recent decades thanks to diterpene glycosides, called steviosides, which it produces. These compounds are characterised by their sweetness, which is 250–300 times higher than saccharose, and they contain almost no caloric value. Stevia is currently also grown outside the South American continent, in various countries characterised by warm weather. This research aimed to determine whether it is viable to grow *Stevia rebaudiana* plants in Poland, a country characterised by a cooler climate than the native regions for stevia plants. Additionally, the impact of adding various dosages and forms of nitrogen fertiliser was analysed. It was determined that *Stevia rebaudiana* grown in Poland is characterised by a rather low concentration of steviosides, although proper nitrogen fertilisation can improve various characteristics of the grown plants. The addition of 100 kg or 150 kg of nitrogen per hectare of the field in the form of urea or ammonium nitrate increased the yield of the stevia plants. The stevioside content can be increased by applying fertilisation using 100 kg or 150 kg of nitrogen per hectare in the form of ammonium sulfate. The total yield of the stevia plants grown in Poland was lower than the yield typically recorded in warmer countries, and the low concentration of steviosides in the plant suggests that more research about growing *Stevia rebaudiana* in Poland would be needed to develop profitable methods of stevia cultivation.

## 1. Introduction

*Stevia rebaudiana* Bertoni, commonly known as ‘stevia’, is a perennial shrub native to the highlands of northeastern Paraguay in South America. This plant has garnered significant attention in recent years due to its natural sweetness [1,2]. The rise in popularity of stevia is attributed to its remarkable sweetness, several times greater than that of sucrose, with the added advantage of low-calorie content. Stevia has emerged as a promising natural substitute for synthetic sweeteners, which can explain the interest in using this plant in the production of various sugar-free products [2,3].

The sweet taste of the stevia leaves derives from so-called steviol glycosides, primarily stevioside and rebaudioside B, which are characterised by 250–300 greater sweetness than sucrose [1,4]. 

Due to the fact that steviol glycosides can be successfully used as a low-calorie sweetener, there is a growing interest in the cultivation of stevia worldwide outside of South America. This has been attempted in China, Malaysia, Singapore, South Korea and Thailand [3].

Furthermore, stevia plants are not only useful in food production but can also be used by the pharmaceutical and cosmetic industries. Steviosides have a positive influence on the human circulatory system as they decrease blood pressure [1,4]. Steviol, a diterpene found in *Stevia rebaudiana* plants, decreases glucose absorption through the small intestines, reducing caloric intake, which can be beneficial for overweight people [5]. Consumption of stevia can prevent the pH level of gastric acid from dropping too low, decrease stomach pains, and reduce the possibility of contracting peptic ulcer disease as well as acid indigestion [6]. Recent studies on the biosynthesis of silver nanoparticles using extracts from *Stevia rebaudiana* have shown that it is possible to acquire novel antibacterial preparations, which could be used as an alternative for antibiotics against Gram-positive and Gram-negative bacteria [7]. Additionally, due to the large amount of phytoconstituents, vitamins and phenolics, *Stevia rebaudiana* can be an active ingredient in various cosmetic products [8,9].

Cultivating stevia in the country where the stevia plants are used could reduce costs and carbon emissions connected with shipping dried stevia plants to different continents [10]. Additionally, the cultivation of stevia nearby the companies using stevia glycosides could help regulate the quality of stevia products and improve sales, as consumers prefer locally sourced food products [11,12,13,14,15].

Stevia is typically grown in warmer regions than most European countries, which is why exploring its growth in cooler climates presents a novel avenue for agricultural research. This study analyses the feasibility of cultivating *Stevia rebaudiana* in Poland, a region characterised by a cooler climate than the plant’s conventional habitats and aims to determine which nitrogen fertilisation treatment could be optimal for the growth of *Stevia rebaudiana* in the Polish climate. Typically grown in tropical and subtropical regions, the adaptation of stevia to a cooler climate present in Poland could yield unique data about the properties of the stevia plant, its biometric characteristics and the yield of the plantation. Nitrogen fertilisation of stevia is a crucial aspect of its cultivation, as adequate nitrogen uptake can influence the final concentration of secondary metabolites in the plant tissue. Nitrogen is also essential in the synthesis of amino acids, proteins, various enzymes and nucleic acid, which are necessary for proper growth of the plant [16,17]. Studies have also shown that increased nitrogen fertilisation during the growth *Stevia rebaudiana* can affect the total yield of the plant [16,17]. In this study, not only was the dosage of nitrogen fertilisation analysed, but different forms of nitrogen fertiliser were also used. The experiment was conducted over three consecutive years in the field. Urea, ammonium nitrate and ammonium sulphate were used as a fertiliser in doses equal to 50, 100 and 150 kg of nitrogen per hectare. The study aimed to analyse yield and various characteristics of *Stevia rebaudiana*, such as plant height, width, number of shoots and concentration of variety of substances in the stevia leaves: elements such as calcium, potassium, magnesium and phosphorus; content of chlorophyll and carotenoids; concentration of ascorbic acid, phenolic compounds, reducing sugars and nitrate ions, as well as concentration of most important glycosides, such as stevioside and rebaudoside A.

## 2. Results

### 2.1. Weights of the Stevia rebaudiana Plants and Their Constituents Grown Using Different Nitrogen Fertilisation Treatments

After harvesting the *Stevia rebaudiana* plants, all the plants were weighed, and then their constituents (leaves, shoots, and plant waste) were weighed. The results of this analysis are presented in Table 1. 

The average weight of the stevia plant was 0.206 kg. U50, U100, U150, N100 and N150 were characterised by greater weight than the average. The highest weight of the stevia plant was measured in the U100. C was characterised by the lowest weight, which was 33% lower than the average weight assessed for all the samples. U50, U100, U150, N100 and N150 were characterised by a leaf weight higher (0.132–0.149 kg) than the average (0.123 kg). The control sample also had the lowest leaf weight: just 62% of the average leaf weight of all the stevia samples analysed in the study. The average shoot weight of stevia plants was equal to 0.073 kg, and the lowest weight was, once again, determined in C (0.056 kg). N150 (0.086 kg), U50 (0.077 kg), U100 (0.088 kg), and U150 (0.084 kg) had shoot weights higher than average. The weight of the plant waste (dried and sick leaves) was greatest in S150, N50 and U50, while it was lowest in C. Data about the weight of all the plants and various parts of the plant material were then used to calculate the percentage that a particular part of the plant constituted in the whole plant material gathered from the experimental field. These data are presented in Table 2. 

Leaves constituted 55.68% of the total weight of C, while in N100, N150, S50, S100, U100, and U150, the ratio of leaf weight to plant weight was above 60%, ranging from 60.11 to 62.62%, with the highest ratio observed for N100. The percentage of shoot weight was greatest for C (40.50%) and lowest for N100 (33.27%). The ratio of plant waste in total weight of the plant ranged from 2.84% to 8.55%. The lowest percentage of plant waste was found in U100, while the highest was observed in S150 and N50. Data from Table 1 and the total area of the experimental field were used to calculate the yield of stevia plants and the yield of particular parts of stevia plants (leaves, shoots and plant waste), as shown in Table 3.

Yield of stevia plants ranged from 13,370 kg·ha^−1^ to 24,370 kg·ha^−1^. The lowest yield was calculated for C, while the highest was for U100. Similarly, the lowest yield of stevia leaves was found for C, and the highest yield of stevia leaves was determined for U50, U100, U150, N150 and N100. Unsurprisingly, the same pattern was observed for the yield of stevia shoots. Regarding the yield of stevia plant waste, C was once again characterised by the lowest yield, but samples with the greatest yield changed by a small margin, as the S150, N50, and U50 were characterised by the greatest yield of plant waste.

### 2.2. Biometrics of the Stevia rebaudiana Grown Using Different Fertilisation Treatments

The basic biometrics of *Stevia rebaudiana* plants, such as the total height of the plant, lateral width of the plant at the level of two-thirds of plant height, number of shoots and number of leaves on the lowest lateral shoot, were measured during the day of the harvest. The results of these analyses are presented in Table 4.

The average height of the *Stevia rebaudiana* plants analysed in this study was equal to 56.63 cm, but almost all fertilised plants were higher than that. The height of fertilised plants ranged from 56.06 cm to 59.50 cm, with the lowest for N50 and the highest for U50, while the height of C was 46.42 cm. C was characterised by the narrowest lateral width of 26.61 cm. Samples fertilised with urea (U50, U100 and U150) were characterised by the widest lateral width (31.06–32.39 cm). C and N100 had the smallest number of shoots (8.44 and 8.72, respectively), which was much smaller than the higher number of shoots in the samples, determined for S50 (13.61), U50 (13.11) and U100 (13.06). The average number of leaves on the lowest lateral shoot was equal to 29.2. C was characterised by the smallest number of leaves on the lowest lateral shoot (22.67). The greatest number of leaves was in plants fertilised with ammonium nitrate (N150), with over 34 leaves on a shoot.

### 2.3. Dry Mass and Concentration of Elements in Stevia rebaudiana Grown Using Different Fertilisation Treatments

The concentration of dry mass in *Stevia rebaudiana* leaves was assessed twice, once during July and once at the harvest, during September. Data are shown in Table 5.

The average dry mass content in the stevia leaves in July was 23.98%, while in September, it increased by 4.80% to 28.78%. The concentration in July ranged from 22.97% for U100 to 26.10% for N100, while the average dry mass content in September was 28.78%. The increase in dry mass content between July and September ranged from 2.88% to 6.15%, with the smallest increase noted for N100 and the largest for N50. 

In stevia leaves during the same periods (July and harvest in September), the concentration of various elements such as calcium, magnesium, phosphorus and potassium was determined. Data about the concentration of particular elements is shown in Table 6.

In most of the analysed samples, the concentration of calcium increased from July to September, with the exception of U50, where it decreased from 887.56 mg·100 g^−1^ d.m. to 863.89 mg·100 g^−1^ d.m. and U150, where it decreased from 1144.44 mg·100 g^−1^ d.m. to 1119.44 mg·100 g^−1^ d.m. The highest concentration of calcium in stevia leaves in July was detected in U150 and the lowest in C (770.89 mg per 100 g of dry mass), while the highest concentration of calcium in September was found in N150, S150 and U150 (1119.44–1188.44 mg·100 g^−1^ d.m.). The concentration of magnesium in the analysed samples also increased from July to September. In July, the concentration of magnesium in the samples ranged from 152.78 mg·100 g^−1^ d.m. (U150) to 183.33 mg·100 g^−1^ d.m. (U50). In September, magnesium content ranged from 214.44 mg·100 g^−1^ d.m. in U50 to 259.44 mg·100 g^−1^ d.m. in S50. The phosphorus content in the samples gathered in September was greater than the phosphorus content in July. The lowest concentration of phosphorus in July was determined for S150 (100.72 mg·100 g^−1^ d.m.), while the highest was determined for N100 (146.28 mg·100 g^−1^ d.m.). In September, U50 was characterised by the greatest concentration of phosphorus (218.77 mg·100 g^−1^ d.m.), while the lowest content of phosphorus in September was detected in U150 (131.13 mg·100 g^−1^ d.m.). The concentration of potassium increased in the samples from July to September. In July, the lowest concentration of potassium was detected in S150 (2275 mg·100 g^−1^ d.m.), while the highest was determined in N50 (2802.78 mg·100 g^−1^ d.m.). In September, S150 was still characterised with the lowest concentration of potassium (2534.78 mg·100 g^−1^ d.m.), while the highest concentration of potassium was detected in S50 (3216.78 mg·100 g^−1^ d.m.). Additional analyses were performed, and during the same periods (July and September), the concentration of ascorbic acid, phenolic compounds, reducing sugars and nitrate ions in stevia leaves was determined. Data about the concentration of these particular components is shown in Table 7.

### 2.4. Concentration of Ascorbic Acid, Phenolic Compounds, Reducing Sugars and Nitrate Ions in Stevia rebaudiana Grown Using Different Fertilisation Treatments

The lowest concentration of ascorbic acid in July was determined for S150, N150 and C (109.27–111.12 mg·100 g^−1^ f.m.), while the highest was for U50. The concentration of ascorbic acid in September was higher than in July for all the samples. The greatest concentration of ascorbic acid in September was detected in S150 (149.28 mg·100 g^−1^ f.m.), and the lowest was in C and N150 (135.42 mg·100 g^−1^ f.m. and 135.89 mg·100 g^−1^ f.m., respectively). U100 was characterised by the greatest concentration of phenolic compounds in July (346.07 mg·100 g^−1^ f.m.), while the content of these compounds was lowest in S50 (203.07 mg·100 g^−1^ f.m.). In September, the lowest concentration of phenolic compounds was determined in C (170.52 mg·100 g^−1^ f.m.) and U150 (172.43 mg·100 g^−1^ f.m.) and the highest for N150 (348.58 mg·100 g^−1^ f.m.). Most of the samples in July were characterised by very similar reducing sugar content, with the exception of C, which had the highest concentration of 1.07 g·100 g^−1^ f.m. and the lowest (of 0.84 g·100 g^−1^ f.m.) for S150. In September, all the samples were characterised by statistically similar concentrations of reducing sugars, ranging from 1.86 g·100 g^−1^ f.m. in U50 to 2.41 g·100 g^−1^ f.m. in N50. In July, the highest concentration of nitrate ions was determined for U100 (2329.44 mg·kg^−1^ d.m.), while the lowest was noted for C (1501.11 mg·kg^−1^ d.m.). The concentration of nitrate ions in September was statistically similar for all the analysed samples and ranged from 890.33 mg·kg^−1^ d.m. for C to 1372.44 mg·kg^−1^ d.m. for S150. Results of the next analysis of the stevia leaves, concerning the concentration of carotenoids and chlorophylls (A and B), are presented in Table 8. 

### 2.5. Concentration of Chlorophyll and Carotenoids in Stevia rebaudiana Grown Using Different Fertilization Treatments

The lowest concentration of chlorophyll A in July was detected in N50 (0.66 mg·g^−1^ f.m.), while the highest was detected in U50 (0.98 mg·g^−1^ f.m.). Most samples gathered in September were characterised by a lower content of chlorophyll A, with the lowest detected in N50 (0.66 mg·g^−1^ f.m.) and highest in U100 (0.86 mg·g^−1^ f.m.). Samples gathered in July were characterised by statistically similar concentrations of chlorophyll B, while in the samples gathered in September, N100 was characterised with the greatest concentration of chlorophyll B (0.51 mg·g^−1^ f.m.), while S100 contains almost two times lower concentration of this compound (0.28 mg·g^−1^ f.m.). The concentration of combined chlorophyll forms (chlorophyll A + B) shows that the average concentration of chlorophylls in July and September was almost the same (1.21 mg and 1.19 mg·g^−1^ f.m.). The concentration of carotenoids was at the same level in all of the samples in July and September.

### 2.6. Concentration of Steviosides in Stevia rebaudiana Grown Using Different Fertilisation Treatments

The final analysis conducted in this study determined the concentration of steviosides (in the form of stevioside and rebaudioside A). The results of this analysis are shown in Table 9.

The concentration of stevioside in the stevia leaves ranged from 108.91 mg to 262.36 mg per 100 g of stevia leaves dry mass, with an average of 156.86 mg. The lowest concentration of stevioside was detected in U50, and the highest was detected in S150. The concentration of rebaudoside A was significantly greater than the concentration of stevioside. On average, the analysed samples contained 998.57 mg·100 g^−1^ d.m. of rebaudoside A, with the greatest concentration detected in S150 (2186.62 mg·100 g^−1^ d.m.) and the lowest in C (352.85 mg·100 g^−1^ d.m.).

### 2.7. Principal Component Analysis of the Data Gathered throughout the Study

**Figure 1:**
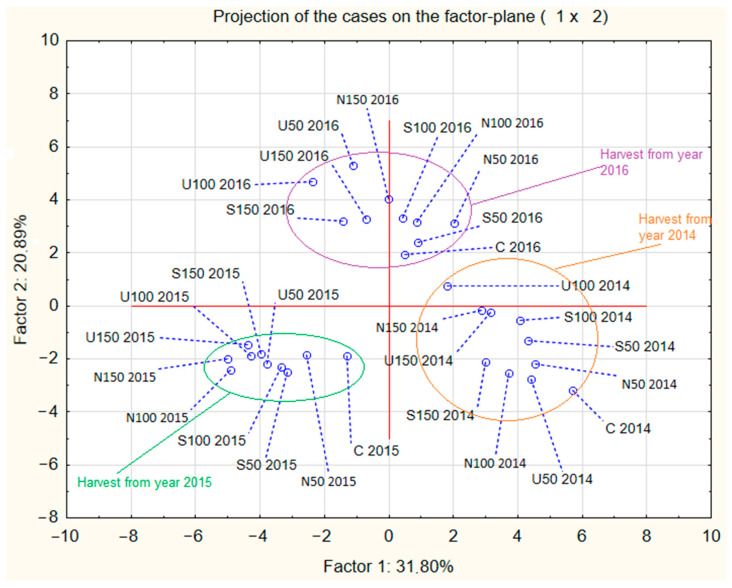
Projection of results of PCA analysis for the analysis of particular characteristics and composition of *Stevia rebaudiana* plants grown in Poland using different nitrogen fertilisation regimes. Abbreviations are as follows: C—stevia plants grown without the addition of nitrogen fertiliser; N50, N100, N150—stevia plants grown with the addition of 50/100/150 kg of N (in the form of ammonium nitrate) per ha of the field; S50, S100, S150—stevia plants grown with the addition of 50/100/150 kg of N (in the form of ammonium sulfate) per ha of the field; U50, U100, U150—stevia plants grown with the addition of 50/100/150 kg of N (in the form of urea) per ha of the field. Numbers 2014, 2015 and 2016 indicate data from the harvest of each particular year.

**Figure 2:**
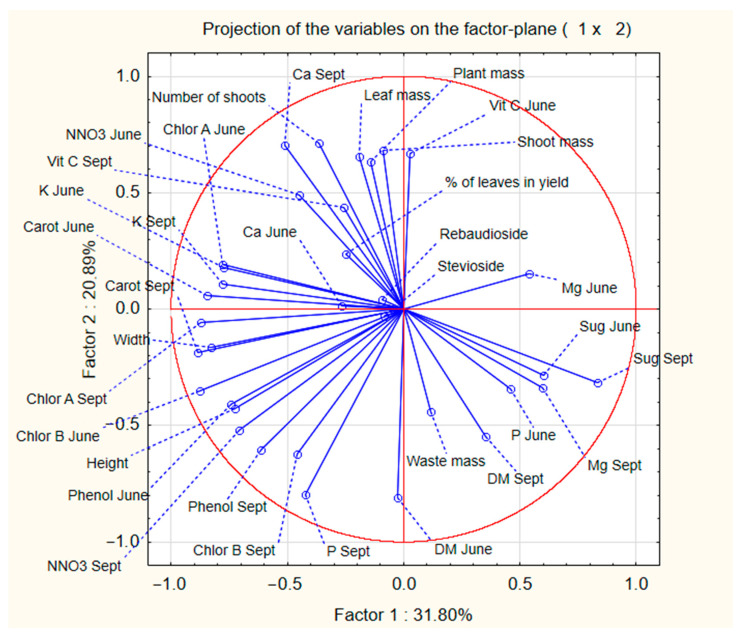
PCA analysis chart for parameters analysed in *Stevia rebaudiana* plants grown in Poland using different nitrogen fertilisation treatments. Abbreviations are as follows: Sept—data from leaves gathered in September. June—data from leaves gathered in June. Ca—calcium content in leaves. Carot—carotenoid content in leaves. Chlor A/B—concentration of chlorophyll A or B. K—potassium content in leaves. DM—dry mass of the stevia leaves. Mg—magnesium content in leaves. NNO3—concentration of nitrate ions in leaves. P—phosphorus content in leaves. Phenol—concentration of phenolic compounds in leaves. Sug—concentration of reducing sugars in stevia leaves. Vit C—concentration of ascorbic acid in leaves.

Principal component analysis (PCA) was conducted on all the mean values of the *Stevia rebaudiana* characteristics gathered throughout the study. The results of the PCA are presented in Figure 1 and Figure 2. The main goal of the PCA analysis was to determine whether the main differences between analysed samples were due to the different fertilisation treatments or due to the year in which stevia plants were planted, as well as to identify any clear influences of various characteristics on other parameters in the analysed samples. 

The results of PCA analysis indicated that the year of harvest was more significant than the type of fertiliser used during the cultivation of *Stevia rebaudiana*. Total plant mass showed a positive correlation with the total mass of shoots and leaves but had a negative correlation with the total waste mass and concentration of dry mass. Plant height had an influence on the concentration of phenols, as the two parameters correlated positively. Few of the analysed parameters significantly influenced the total content of stevioside and rebaudoside A in stevia plants. 

## 3. Discussion

### 3.1. Weight of the Stevia rebaudiana Plants and the Yield of the Cultivation of Stevia Plants

The acquired data shows that the plants grown on the fertilised field yielded a greater mass of stevia plants than the unfertilised sample (C) and were characterised by the highest yield of leaves, which are the primary reason for *Stevia rebaudiana* plantation. This finding is confirmed by various other researchers, such as Mahajan and Pal (2022), Sun et al. (2020) and Inugraha et al. (2014) [18,19,20]. These results are rather typical, as nitrogen is an element which can significantly impact plant metabolism and is one of the key components during all plant growth stages [21]. It was also unsurprising to find that the weight of the plants and shoots in samples fertilised with the highest dosage of nitrogen was also the highest for the same reason. The yield of the stevia plants was not far lower than the yield of *Stevia rebaudiana* planted in the warmer climate of Ethiopia, which ranged from 16,951 to 26,388 kg·ha^−1^, depending on the spacing between the plants [22]. However, it is necessary to acknowledge the fact that research conducted in Ethiopia did not involve any nitrogen fertilisation. Therefore, it would be more appropriate to compare this data to C, which was characterised by a lower yield of 13,770 kg·ha^−1^. On the other hand, data from the experiment conducted by Shivani et al. (2019) in India, a country in which the stevia plants are commercially grown, at a latitude closer to its natural habitat (25°39′42′’ N compared to 19°–27° S of Paraguay) than Poland (49°-54°50′ N, with Psary and Wroclaw at ~51.06° N) shows, that the yield of stevia leaves was equal to 5150–7040 kg/ha (dependent on the plant spacing) with the nitrogen fertilisation of 50 kg N per ha and 6280–10,540 kg·ha^−1^ with the nitrogen fertilisation of 100 kg N per ha [23]. Data acquired in the study in Poland shows that it is possible to acquire an even greater yield of stevia leaves using 50 kg of N·ha^−1^ than using 100 kg of N per ha in India. Additionally, the so-called ‘harvest index’ (ratio of leaves to the weight of the plant) of these plants was in the range of 39.33–43.67%, which was much lower than in our study. These data indicate that the amount of harvested plant material of *Stevia rebaudiana* grown in Poland could be on a similar level as in various other countries. The main differences in the stevia yield in Poland and in various other countries are most probably dictated by the large differences in temperature, as well as rainfall at these places (Table 10 and Table 11). In Paraguay, temperatures of the months equivalent to the May-September period in the northern hemisphere (November-March) are between 27.2–29.6 °C, while in Poland, the average temperature in these months during the time of the study was in the range of 13.4–25.2 °C, which is significantly lower [24,25]. Data gathered by Warner (2022) have shown that stevia plants grown at a temperature of 26 °C, compared to the stevia plants grown at 17 °C were characterised by twice the height, almost three times longer branches and two times greater development rate (nodes per day), suggesting that the relatively low yield of the stevia grown in Poland might be due to lower temperatures [26]. Similarly, Paraguay receives a greater amount of rainfall during the months of stevia cultivation than Poland (116 mm of rainfall monthly compared to 52 mm of rainfall monthly during the study period) [24,25]. However, due to irrigation during periods of drought, the differences in the amount of available water might not be as crucial as the differences in the temperature during the growth of stevia.

### 3.2. Biometrics of the Stevia rebaudiana Grown Using Different Fertilisation Treatments

It is common knowledge that proper nutrition of growing plants contributes to the increase in plant size and number of shoots and leaves, crucial parameters influencing the yield of herbaceous plants [27,28,29,30,31]. The conducted experiment demonstrates that the addition of just 50 kg of N per hectare can increase the height of the stevia plants by over 20% and appears necessary for cultivating stevia in Poland. However, plant height is not as important as the number of shoots and leaves, as leaves are the primary goal of stevia plantations. Additionally, thanks to the PCA analysis, it can be observed that while nitrogen addition did not consistently increase the number of shoots (C and N100 had statistically similar shoot levels), fertilisation enhanced the number of leaves on the lowest lateral shoots (the longest and oldest shoots of the plant, typically with the largest leaves) [32]. Interestingly, the amount of fertiliser used did not have as much influence on the various biometrics of stevia as the type of fertiliser used. Plants fertilised with ammonium nitrate were characterised with a lower number of shoots but a greater amount of leaves on the shoots than plants fertilised with ammonium sulfate or urea. Unfortunately, data from this study does not provide insight into the reasons for this observation, but this information can be an interesting avenue for future research. An experiment performed by Tadesse, mentioned earlier in Section 3.1, about the plantation of stevia in Ethiopia also measured various biometrics of stevia plants [22]. Plants grown in Ethiopia were characterised by lower plant height than stevia plants grown in Poland, which allowed for the acquisition of greater yield. This suggests that the increase in height of fertilised stevia plants analysed in this study might not necessarily be an advantage over C. However, another experiment performed by Baghat et al. in Jammu, India (32°74′ N) also showed that plants fertilised with the addition of nitrogen were characterised with 13–38% greater height than the unfertilised control sample and a far greater amount of leaves (by 17–80%) [33]. Therefore, it seems reasonable to assume that fertilisation of *Stevia rebaudiana* plants with nitrogen might result in taller stevia plants, but most certainly will result in a greater amount of plant leaves, which are the main goal of stevia plantation and, therefore, would be recommended practice for the farmers.

### 3.3. Dry Mass and Concentration of Elements in Stevia rebaudiana Grown Using Different Fertilisation Treatments

The dry mass content in the leaves of stevia increased from July to September in all the samples. There appeared to be no difference in the dry mass content in leaves of stevia plants grown with the addition of nitrogen compared to those grown without it. Furthermore, the amount and type of fertiliser used did not significantly impact the dry mass content of the stevia leaves. However, the concentration of various elements (calcium, magnesium, phosphorus and potassium) differed significantly between the analysed samples. Both the fertilisation treatment and the time of the sample analysis (July and September) influenced the concentration of various elements. The concentration of each analysed element in September was higher than in July, which might be one of the factors influencing changes to the dry mass content of the stevia leaves. The concentration of calcium in the dried stevia leaves, which were gathered from the plants grown in La Serena, Chile (29°54′ S), was equal to 1388.37 mg, which was 20–40% higher than the concentration of calcium in the samples grown in this study in Poland [34]. A different batch of stevia leaves, analysed by Tadhani and Subhash, grown in India in an undisclosed location, was characterised by a concentration of calcium closer to 1500 mg [35]. A comprehensive review by Lemus-Mondaca has shown that the concentration of calcium in stevia leaves ranges typically from 500 to 1500 mg per 100 g of dry leaves, indicating that the concentration of calcium in the stevia leaves grown in Poland contains an average concentration of calcium during September and rather low concentration of this element in July [36]. It should be mentioned that increasing the amount of fertiliser used always increased calcium concentration in the leaves, regardless of the type of fertiliser used or whether the leaves were analysed in July or September. The concentration of magnesium in stevia leaves, with an average of 232.56 mg per 100 g of leaf dry mass in September, was far lower than the content of stevia leaves analysed by Tadhani and Subhash (~500 mg) and by Lemus-Mondaca et al. (2016) (867.79–1013.30 mg∙100 g^−1^ d.m.) [34,35]. The review of various *Stevia rebaudiana* samples has shown that the magnesium content in stevia leaves can be as low as 349 mg∙100 g^−1^ d.m., which is still higher than the magnesium content of leaves of stevia grown in Poland. The low concentration of magnesium in stevia leaves might be an indicator of various problems concerning *Stevia rebaudiana* cultivation in Poland, as it has been shown that magnesium content and bioavailability can relate to the production of steviol glycosides (with the high concentration of magnesium correlating positively with the concentration of steviosides) [36,37,38]. The concentration of phosphorus in stevia plants grown in this study was also lower (123.97 mg∙100 g^−1^ d.m. in July, 168.34 mg∙100 g^−1^ d.m. in September) than the phosphorus content in the Tadhani and Subhash stevia samples (~350 mg) [35]. However, authors like Ucar et al. (2018) determined that the concentration of phosphorus in stevia leaves, gathered from stevia grown with the addition of 80 kg of N·ha^−1^ in Antalya, Turkey (36°54′ N), was characterised by phosphorus content of 120 mg∙100 g^−1^ d.m. in July and 190 mg∙100 g^−1^ d.m. in September [39]. However, the concentration of potassium in the samples of Ucar et al. (2018) was in the range of 1300–1400 mg∙100 g^−1^ d.m., which is over two times lower than the concentration of potassium in the stevia grown in Poland. The concentration of potassium in the aforementioned analysis of Tadhani and Subhash was at a similar level (2510 mg∙100 g^−1^ d.m.) as the concentration of potassium in samples gathered in Poland [35]. Potassium is one of the elements necessary for the synthesis of glycosides, and a low concentration of potassium can reduce the number of steviol glycosides by 15–25%, but potassium-deficient stevia leaves were characterised by a potassium content of ~1500 mg, while non-deficient stevia was characterised by a potassium content of 2000 mg [40]. This shows that stevia plants grown in this study in typical conditions in Poland are rather not deficient in potassium, which indicates a perspective for the cultivation of this plant in Poland.

### 3.4. Concentration of Ascorbic Acid, Phenolic Compounds, Reducing Sugars and Nitrate Ions in Stevia rebaudiana Grown Using Different Fertilisation Treatments

Ascorbic acid is one of the compounds responsible for the anti-inflammatory and antioxidant properties of *Stevia rebaudiana* leaves [8,9]. The concentration of ascorbic acid increased from July to September in all the stevia samples. Stevia plants grown in this study were characterised by similar concentrations of ascorbic acid as samples analysed by Lemus-Mondaca and 10 times larger concentration than in the study conducted by Kim et al. [41]. The high concentration of ascorbic acid in stevia plants is of interest due to its synergistic effect with stevia polyphenols, which significantly increases stevia’s antioxidant potential. Changes in the concentration of phenolic compounds from July to September in the stevia leaves were not as clear as the changes in the ascorbic acid content, as some samples were characterised by the greatest concentration of phenolic compounds in July, some in September, and in some samples, the concentration of these components remainder relatively the same. However, the concentration of phenolic compounds in stevia leaves from the study of Lemus-Mondaca was far lower (up to even three times) than the concentration of phenolic compounds in stevia leaves analysed in this study, suggesting that, with the relatively high concentration of ascorbic acid, stevia plants grown in Poland might be characterised with far greater antioxidant and anti-inflammatory properties than stevia grown in warmer climate [41]. However, more research about this hypothesis would have to be conducted in the future. The level of reducing sugars increased in stevia leaves between July and September; however, it is a known mechanism present in various plants from a variety of families [42,43,44,45]. The content of nitrate ions in the plant tissues is an indicator of the nutrient status of the plant, as the uptake of nitrate ions is related to the concentration of nitrates in the soil and the plant’s ability to assimilate these ions. As the stevia plants were fertilised at the start of their growth, it is not surprising to see that the concentration of nitrates in September is far lower than in July. This indicates that stevia plants have used some of the reserves of nitrate, which were assimilated when the plant was young [46,47]. Interestingly, N100 and N150, as well as S100 and S150, were characterised with a similar concentration of nitrate ions in July and U150 was characterised with a lower concentration of nitrate ions than U100. Therefore, it seems that the young *Stevia rebaudiana* plants are not able to sufficiently use nitrogen from the dosage of 150 kg of N per hectare. Thus, it is possible that the highest concentration of nitrogen, which should be applied to the *Stevia rebaudiana* plantation in order to increase nitrate concentration, should be 100 kg of N per ha or less. 

### 3.5. Concentration of Chlorophyll and Carotenoids in Stevia rebaudiana Grown Using Different Fertilization Treatments

The concentration of chlorophyll remained at a rather similar level in most of the samples analysed during July and September. It is important to note that the concentration of chlorophyll in plant leaves can be a useful indicator of the development of chloroplasts, photosynthetic capacity and overall plant vigour [48]. The concentration of chlorophyll A in the stevia leaves was higher than the concentration of chlorophyll B, as is typical in most plants [49,50,51,52]. The concentration of chlorophyll A in July was at a similar level in most of the samples, with the exception of the sample with the lowest content (N50) and the highest content (U50). This fact might suggest that the amount of fertiliser is not as important when it concerns the concentration of chlorophyll, but the form of the nitrogen introduced to the plants might have a more important role. However, interestingly, N100, measured in September, was characterised by the greatest concentration of chlorophyll B, while S100 was characterised by the lowest. The data acquired in this research does not provide precise answers for this phenomenon, but these results could be analysed more meticulously in future studies. It is important to note, however, that stevia plants analysed in this study were characterised by rather low (3–4 times lower) concentration of chlorophyll when compared to the data acquired by Shahverdi et al. (2019), grown in Karaj Iran (35°48′ N) [53]. Similar differences can be seen between samples of Ucar et al. grown in Antalya, Turkey (36°54′ N), where the total concentration of chlorophyll in stevia leaves was equal to 7.5 mg∙g^−1^ of fresh leaf mass (concentration six times higher than in Polish stevia plants) [39]. These results suggest that the impact of nitrogen fertilisation is minuscule when compared to the impact of the amount of sunlight that the stevia plant acquires during its growth. Carotenoid content in the stevia leaves was not affected by the changes in nitrogen fertilisation and remained at almost the same level in July and September. The concentration of carotenoids in plant leaves is often related to the intensity of light during growth of the plant, as demonstrated in the study by Simlat et al. (2016) in *Stevia rebaudiana* plants [54]. This fact might explain the lower concentration of carotenoids in stevia plants grown in Poland when compared to stevia plants grown in Iran, which were characterised by a 3–4 times higher concentration of carotenoids in leaves [55].

### 3.6. Concentration of Steviosides in Stevia rebaudiana Grown Using Different Fertilisation Treatments

Steviosides are the primary reason for cultivating *Stevia rebaudiana* plants, and the goal of stevia farmers is to achieve the largest yield with the highest concentration of steviosides [56]. The main factors influencing stevioside content are temperature and amount of sunlight received by the plants, as well as the variety of the *Stevia rebaudiana* plant [57,58]. Stevia plants analysed in this study exhibited a very low concentration of steviosides compared to those grown in warmer countries, where the concentration typically ranges from 4% to even 20% of the dry weight [59,60]. However, one of the crucial aspects of this work is the fact that the addition of nitrogen fertiliser increased the total concentration of steviosides in the stevia plants grown in Poland. Additionally, it is worth noting that S100 and S150 were characterised with far greater concentrations of steviosides than other samples, indicating that this type and amount of fertiliser should be used by farmers seeking to maximise the concentration of rebaudioside A and stevioside in stevia plants grown in Poland. Nevertheless, it is crucial to point out that one of the most important factors influencing the concentration of steviosides in *Stevia rebaudiana* leaves is the temperature at which the plants are growing [61,62]. As mentioned in Section 3.1., Poland experiences much lower temperatures than regions typically suited for stevia growth. Higher temperatures of the stevia cultivation, with the optimum close to 25 °C, have been shown to result in higher transcript levels of fifteen steviol glycoside biosynthesis pathways genes, while the temperatures close to 15 °C (similar to the average temperature of May, June and September in Poland) significantly restrain the transcription of these genes [61,62]. 

## 4. Materials and Methods

### 4.1. Experimental Design

#### 4.1.1. Preparation of Seedlings

Seedlings used in the study were grown in a heated greenhouse. Seeds of the *Stevia rebaudiana* plant (Legutko Seed Company, Jutrosin, Poland) were sown during 1st decade of April of the year 2014, 2015, and 2016 in seed trays (30 × 40 cm) filled with peat substrate (Compo Sana) which were then covered with the thin layer of peat substrate and watered. After 3–4 weeks from seed sowing (during 1st decade of May), seedlings with at least one pair of foliage leaves were transferred to 54-site propagation kits filled with peat substrate. Later, during 3rd decade of May, plants with at least three pairs of foliage leaves and a height of at least 10 cm were transferred to pots with a diameter of 10 cm. Prior to adding peat substrate, the seed trays, propagation kits and pots were disinfected with 1% solution (*v*/*v*) of HortiSept 402D (Hortico, Wrocław, Poland).

#### 4.1.2. Preparation of the Field Used to Perform the Experiment

Prior to transplanting the seedlings from the greenhouse to the field, specific agrotechnical procedures were carried out on the field. In the winter preceding the experiment (i.e., 2013 for the experiment commencing in 2014, etc.), the field underwent ploughing. During the spring of the experiment year, the field was further prepared by dragging, tilling and harrowing. The soil selected for the experiment was black degraded soil with a humus content of 1.8%, slightly alkaline pH and salinity of 57 μS·cm^−3^. The soil’s phosphorus concentration was 41 mg·dm^−3^; potassium concentration was 320 mg·dm^−3^; calcium concentration was 3000 mg·dm^−3^; and magnesium concentration was 30 mg·dm^−3^. Additionally, the soil contained a trace amount of NO_3_^−^ ions. After applying pre-plant fertilisation according to the methodology described in Section 4.1.3, the fertiliser was incorporated into the soil, and the field was covered with black agro-textile (UV P-100) to suppress weed growth. Additional fertiliser in the form of ammonium nitrate (50 kg N·ha^−1^) was applied as a top-dressing four weeks after planting the seedlings. 

#### 4.1.3. Field Experiment

Field experiment involving *Stevia rebaudiana* plants was conducted in the years 2014, 2015 and 2016 at the Research and Didactic Station in Psary, Wroclaw University of Environmental and Life Sciences: (51°19′055913609418″ N, 17°03′36781036313″ E). The experiment followed a split-plot design with three replications. Two main factors were considered throughout the experiment: fertiliser amount (three different doses) and fertiliser type (three different nitrogen forms: NH_4_NO_3_, (NH_4_)_2_SO_4_, CH_4_N_2_O) used for pre-plant fertilisation. The fertiliser was thoroughly mixed with the soil prior to planting the *Stevia rebaudiana* seedlings. This experimental setup allowed for the investigation of ten different fertilisation treatments for *Stevia rebaudiana*:

- Control plots (C): no fertiliser was applied before planting the seedlings.
- Plots fertilized with ammonium nitrate (50 kg N·ha^−1^) before planting the seedlings (N50)
- Plots fertilized with ammonium nitrate (100 kg N·ha^−1^) before planting the seedlings (N100)
- Plots fertilized with ammonium nitrate (150 kg N·ha^−1^) before planting the seedlings (N150)
- Plots fertilized with ammonium sulfate (50 kg N·ha^−1^) before planting the seedlings (S50)
- Plots fertilized with ammonium sulfate (100 kg N·ha^−1^) before planting the seedlings (S100)
- Plots fertilized with ammonium sulfate (150 kg N·ha^−1^) before planting the seedlings (S150)
- Plots fertilized with urea (50 kg N·ha^−1^) before planting the seedlings (U50)
- Plots fertilized with urea (100 kg N·ha^−1^) before planting the seedlings (U100)
- Plots fertilized with urea (150 kg N·ha^−1^) before planting the seedlings (U150)

In total, the experiment comprised 30 field plots, each with an area of 1 m^2^. Ten stevia seedlings (as described in Section 4.1.1) were planted in each plot with a spacing of 45 cm × 20 cm. Each field plot was separated from adjacent plots by an uncultivated soi strip with a width of 50 cm. The total area of the experiment was 62.5 m^2^. Standard agronomic practices were followed for plant care, including manual weed removal and irrigation during dry periods with 20 mm of water per irrigation session. Temperature and rainfall data during the experiment period at the stevia planting site are presented in Table 10 and Table 11.

#### 4.1.4. Harvesting

The *Stevia rebaudiana* plants were harvested in the first decade of September each year, before the flowering stage. Harvesting was conducted manually by cutting the stalk 10 cm over the ground. During harvesting, the plants were weighed to determine the total biomass yield per unit area. After weighing, the leaves, shoots and any plant waste (sick or dried leaves) were weighed separately. These data were then used to calculate the percentage of leaves, shoots and plant waste in the harvested plant material. A total of ten measurements were taken each year (one per plant), resulting in 30 measurements for the entire experiment. The yield was calculated based on three measurements (once per year).

#### 4.1.5. Biometrics Measurements

Before harvesting, simple biometric measurements were taken on fully developed and healthy plants. The total height of the plant was measured, as well as lateral width at two-thirds of the plant height. The number of first-order shoots and the number of leaves on the lowest lateral shoot were recorded. Six measurements were taken each year, resulting in 18 measurements for the entire experiment.

### 4.2. Chemical Analyses

All analyses were conducted on healthy, undamaged leaves of *Stevia rebaudiana* collected from the midpoint of the plant height. Each analysed sample consisted of leaves gathered from six plants from each field, which weighed approximately 10–15 g per plant. Leaves were collected once in July and once at harvest in September. Three measurements were taken each year in July and September, resulting in a total of eighteen measurements for the entire experiment, except for the concentration of steviosides, which was only analysed in September.

#### 4.2.1. Dry Mass Content

The dry mass content of the analysed leaves was determined using the PN-90/A-75101/03 method, utilising a Binder ED400 (Merazet, Poznan, Poland) dryer and a WTC2000 (Radwag, Radom, Poland) analytical scale. Approximately 10–20 g of fresh leaf material was used for each analysis [63].

#### 4.2.2. Extraction of Elements and Ions from the Plant Material

Before analysing the concentration of nitrate ions, magnesium, potassium, calcium and phosphorus, extracts of these elements had to be prepared according to the method by Nowosielski [64]. Dried and ground leaves (0.4 g) were transferred to plastic containers with the screw-cap, to which 100 cm^3^ of 2% (*v*/*v*) acetic acid and 1 g of active carbon were added. The containers were shaken for 30 min. in a MaxQ2000 shaker (Thermo Fischer Scientific, Waltham, MA, USA) at 150 rpm. After shaking, the mixture was filtered through a paper filter to 200 cm^3^ Erlenmeyer flasks. The clear filtrate was then used for subsequent analyses of element and ion concentrations.

##### Concentration of Magnesium in Plant Material

The magnesium content in the filtrate was assessed spectrophotometrically using a WPA S106 spectrophotometer (Biochrom, Cambridge, UK). Five cm^3^ of the filtrate was transferred using an automatic pipette to graduated glass test tubes. Twenty cm^3^ of the compound reagent (consisting of 480 mg sodium hydroxide, 250 mg of glycerol, 50 mg hydroxylammonium chloride, 50 mg polyvinyl alcohol and 1.25 mg of titan yellow, adjusted to 20 cm^3^ with distilled water) was added to the tubes. Then, 3.5 cm^3^ of the resulting solution was transferred to a polystyrene cuvette (optical path of ten mm). Absorbance was read at the wavelength equal to 555 nm. A blank sample was created by adding five cm^3^ of 2% acetic acid solution to the twenty cm^3^ of the previously described compound solution.

##### Concentration of Phosphorus in Plant Material

The phosphorus content in the filtrate was assessed spectrophotometrically. Twenty cm^3^ of the filtrate was transferred to graduated glass test tubes, and 5 cm^3^ of a solution containing 2.5 mg of ammonium metavanadate and 125 mg of ammonium heptamolybdate was added. The resulting solution was transferred (3.5 cm^3^) to a polystyrene cuvette, and absorbance was measured at the wavelength of 470 nm. A blank sample was created by adding twenty cm^3^ of 2% acetic acid solution to five cm^3^ of the solution containing 2.5 mg of ammonium metavanadate and 125 mg of ammonium heptamolybdate.

##### Concentration of Calcium and Potassium in Plant Material

The concentration of calcium and potassium in the plant material was determined in the filtrate using flame photometry with a Carl Zeiss Jena-type flame photometer.

##### Concentration of Nitrate Ions in Plant Material

The concentration of nitrate ions in the plant material was determined in the filtrate using an Orion 5 Star ionometer (Thermo Fischer Scientific, Waltham, MA, USA) calibrated in the range of 10–1000 mg of NO_3_^−^ per dm^3^ of the sample.

#### 4.2.3. Concentration of Reducing Sugars in the Plant Material

Twenty grams of fresh stevia leaves were weighed and transferred to 200 cm^3^ volumetric flasks. Ten cm^3^ of distilled water was added to each flask, and the flasks were placed in a water bath filled with boiling water for 30 min. After this procedure, the concentration of reducing sugars in the plant material was determined using Lane-Eynon general volumetric method accepted by the Polish Committee for Standarisation, described previously by Godlewska et al. (2021) [PN-90/A-75101/07] [65,66].

#### 4.2.4. Concentration of Carotenoids and Chlorophyll A and B

The concentration of chlorophyll A, chlorophyll B and carotenoids was determined spectrophotometrically. Fresh stevia leaves (0.4 g) were weighed and transferred to the mortar, where they were ground with the addition of previously dried sand and calcium carbonate. A few drops of 80% acetone were added to the mortar, and the mortar contents were ground to obtain a homogenous mass. Twenty cm^3^ of acetone was used to transfer the ground leaves to the Schott filtration system and filter them under the vacuum. The filtrate was transferred to a 50 cm^3^ volumetric flask, filled with 80% acetone and mixed thoroughly. The absorbance of the filtrate was measured in the quartz cuvettes (optical path of ten mm) at the wavelengths of 470 nm, 645 nm and 663 nm. The blank sample consisted of 80% acetone [67].

#### 4.2.5. Concentration of Vitamin C (Ascorbic Acid)

The concentration of vitamin C (ascorbic acid) in plant material was determined using methodology by Bieniek (2012) and Krężel and Kołota (2014), as previously outlined by the Polish Committee for Standardization [67,68,69]. Fresh stevia leaves (~10 g) were homogenised using a Koenic blender with oxalic acid (200 cm^3^, 2%). The resulting solutions were filtered, and filtrates (10 cm^3^) were collected and titrated with a solution of 2,6-dichlorophenolindophenol (Tillmans’ reagent) until a light pinkish colour appeared and lasted for at least 1 min [67,68].

#### 4.2.6. Concentration of Phenolic Compounds

The total concentration of phenolic compounds was measured according to the Folin–Ciocalteu method described by Jałoszyński et al. (2008) with slight modifications described by Godlewska et al. (2021) by extracting the phenolic compound from a 5 g sample of stevia leaves [66,70].

#### 4.2.7. Concentration of Steviosides

The concentration of stevioside and rebaudoside A in the plant material was determined using high-performance liquid chromatography as previously described by Vanĕk et al. [71]. The concentration of steviosides was measured only in the final product (leaves gathered in September). Two grams of leaf sample was used for one extraction procedure per year, and HPLC analysis was performed three times on each of the extracts.

### 4.3. Data Analysis

The data collected over all years during this study (biometrics measurements, concentration of various elements and ions in plant material, concentration of reducing sugars, chlorophyll A, chlorophyll B, carotenoids, ascorbic acid, phenolic compounds and steviosides) were statistically analysed using Statistica. A two-way ANOVA (α = 0.05) was employed, with the Duncan test used to establish homogenous groups. Additionally, to determine whether the main differences between the analysed samples were due to different fertilisation treatments or the year in which stevia plants were planted, a principal component analysis (PCA) was conducted on the mean results from each year of the study. In the PCA, the single-linkage agglomerative method was used, employing the Euclidean measure to determine the distance between each analysed parameter and the samples of stevia grown with different fertilisation treatments each year.

## 5. Conclusions

Cultivating *Stevia rebaudiana* plants in Poland, in comparison to countries closer to the equator where temperatures are higher and which receive more sunlight, appears to be less profitable due to lower yields and lower concentrations of steviosides in the plant leaves. However, the addition of various nitrogen fertilisers can enhance the quality and yield of *Stevia rebaudiana* plantations in Poland. The addition of 100 kg or 150 kg of nitrogen per hectare of the field in the form of urea or ammonium nitrate increases the total yield of the stevia plants. Conversely, the addition of 100 kg and 150 kg of nitrogen per hectare of the field in the form of ammonium sulfate does not increase the stevia yield as the addition of mentioned fertilisers, but it maximises the number of steviosides present in the *Stevia rebaudiana* leaves. Data collected thus far indicates that cultivating *Stevia rebaudiana* in Poland presents challenges due to the low concentration of steviosides in the plants, which is the primary goal of stevia cultivation. However, further research into stevia cultivation in Poland could be a worthwhile avenue of study in light of progressive climate change, which is increasing average temperatures in the region.

## Figures and Tables

**Table 1:** Weight of the stevia plants grown with different nitrogen fertilisation treatments.

Sample ^1^	Single Plant Weight [kg]	Plant Leaf Weight [kg]	Plant Shoot Weight [kg]	Plant Waste Weight [kg]
C	0.137 ± 0.059 d	0.077 ± 0.029 e	0.056 ± 0.031 c	0.005 ± 0.008 d
N50	0.180 ± 0.083 c	0.102 ± 0.045 d	0.064 ± 0.032 bc	0.014 ± 0.015 ab
N100	0.211 ± 0.065 abc	0.132 ± 0.045 abc	0.070 ± 0.024 abc	0.009 ± 0.010 bcd
N150	0.243 ± 0.073 a	0.148 ± 0.046 a	0.086 ± 0.031 a	0.009 ± 0.012 bcd
S50	0.183 ± 0.068 c	0.110 ± 0.040 cd	0.065 ± 0.030 bc	0.008 ± 0.010 bcd
S100	0.196 ± 0.080 bc	0.120 ± 0.050 bcd	0.070 ± 0.031 abc	0.006 ± 0.007 cd
S150	0.206 ± 0.081 abc	0.117 ± 0.048 cd	0.072 ± 0.037 abc	0.018 ± 0.016 a
U50	0.221 ± 0.088 abc	0.132 ± 0.059 abc	0.077 ± 0.033 ab	0.012 ± 0.015 abc
U100	0.244 ± 0.075 a	0.149 ± 0.046 a	0.088 ± 0.031 a	0.007 ± 0.007 cd
U150	0.238 ± 0.085 ab	0.146 ± 0.049 ab	0.084 ± 0.037 a	0.009 ± 0.009 bcd
Total average	0.206 ± 0.082	0.123 ± 0.051	0.073 ± 0.033	0.010 ± 0.012

^1^ Values are expressed as the mean (n = 30) ± standard deviation. Mean values with different letters (a, b, c, d, e) within the same column are significantly different (α = 0.05) according to Duncan’s test. Abbreviations are as follows: C—stevia plants grown without the addition of nitrogen fertiliser; N50, N100, N150—stevia plants grown with the addition of 50/100/150 kg of N (in the form of ammonium nitrate) per ha of the field; S50, S100, S150—stevia plants grown with the addition of 50/100/150 kg of N (in the form of ammonium sulfate) per ha of the field; U50, U100, U150—stevia plants grown with the addition of 50/100/150 kg of N (in the form of urea) per ha of the field.

**Table 2:** Percentage of various parts of the yield of stevia plants grown with different nitrogen fertilisation treatments.

Sample ^1^	Leaf Mass Fraction [%]	Shoot Mass Fraction [%]	Plant Waste Fraction [%]
C	55.68 ± 9.85 abc	40.50 ± 9.73 a	3.81 ± 1.75 c
N50	56.94 ± 6.23 bc	35.46 ± 6.31 ab	7.59 ± 4.87 ab
N100	62.62 ± 7.76 a	33.27 ± 6.99 b	4.11 ± 2.35 bc
N150	60.98 ± 5.59 ab	35.39 ± 5.05 ab	3.63 ± 2.53 c
S50	60.11 ± 6.09 ab	35.50 ± 5.61 ab	4.39 ± 2.21 bc
S100	61.12 ± 4.95 ab	35.56 ± 5.00 ab	3.23 ± 1.60 c
S150	56.55 ± 9.92 c	34.90 ± 5.70 b	8.55 ± 4.10 a
U50	59.68 ± 6.57 abc	34.77 ± 6.36 ab	5.55 ± 3.18 bc
U100	61.13 ± 5.90 ab	36.02 ± 5.19 ab	2.84 ± 1.80 c
U150	60.99 ± 6.58 ab	35.26 ± 5.75 ab	3.74 ± 1.46 c
**Total average**	**59.58 ± 7.28**	**35.66 ± 6.36**	**4.74 ± 3.20**

^1^ Values are expressed as the mean (n = 30) ± standard deviation. Mean values with different letters (a, b, c) within the same column are significantly different (α = 0.05) according to Duncan’s test. Abbreviations are as follows: C—stevia plants grown without the addition of nitrogen fertiliser; N50, N100, N150—stevia plants grown with the addition of 50/100/150 kg of N (in the form of ammonium nitrate) per ha of the field; S50, S100, S150—stevia plants grown with the addition of 50/100/150 kg of N (in the form of ammonium sulfate) per ha of the field; U50, U100, U150—stevia plants grown with the addition of 50/100/150 kg of N (in the form of urea) per ha of the field.

**Table 3:** Yield of the stevia plants grown with different nitrogen fertilisation treatments.

Sample ^1^	Yield of Stevia Plant [kg·ha^−1^]	Yield of Stevia Leaves [kg·ha^−1^]	Yield of Stevia Shoots [kg·ha^−1^]	Yield of Stevia Plant Waste [kg·ha^−1^]
C	13,770 ± 2010 b	7630 ± 400 c	5570 ± 1210 c	530 ± 230 c
N50	18,570 ± 2490 ab	10,630 ± 1200 b	6530 ± 1140 b	1400 ± 410 a
N100	21,100 ± 980 a	13,230 ± 310 a	7000 ± 960 a	870 ± 400 ab
N150	24,330 ± 2510 a	14,830 ± 1630 a	8600 ± 1310 a	900 ± 460 ab
S50	18,300 ± 1770 ab	11,030 ± 1010 b	6500 ± 610 b	800 ± 200 b
S100	19,630 ± 3660 ab	12,030 ± 1780 ab	6970 ± 1360 ab	630 ± 120 bc
S150	20,630 ± 3550 a	11,700 ± 2690 ab	7200 ± 1410 ab	1730 ± 620 a
U50	22,130 ± 4950 a	13,200 ± 2270 a	7700 ± 1270 ab	1230 ± 400 a
U100	24,370 ± 4900 a	14,900 ± 2860 a	8770 ± 1060 a	700 ± 170 b
U150	23,870 ± 3610 a	14,530 ± 1530 a	8400 ± 1220 a	870 ± 340 ab
**Total average**	**20,670 ± 4390**	**12,370 ± 2940**	**7320 ± 1700**	**970 ± 690**

^1^ Values are expressed as the mean (n = 30) ± standard deviation. Mean values with different letters (a, b, c) within the same column are significantly different (α = 0.05) according to Duncan’s test. Abbreviations are as follows: C—stevia plants grown without the addition of nitrogen fertiliser; N50, N100, N150—stevia plants grown with the addition of 50/100/150 kg of N (in the form of ammonium nitrate) per ha of the field; S50, S100, S150—stevia plants grown with the addition of 50/100/150 kg of N (in the form of ammonium sulfate) per ha of the field; U50, U100, U150—stevia plants grown with the addition of 50/100/150 kg of N (in the form of urea) per ha of the field.

**Table 4:** Biometrics of stevia plants grown with different nitrogen fertilisation treatments.

Sample ^1^	Plant Height [cm]	Lateral Width [cm]	Number of Shoots	Number of Leaves on the Lowest Lateral Shoot
C	46.42 ± 6.03 b	26.61 ± 4.12 d	8.44 ± 2.09 c	22.67 ± 4.95 c
N50	56.06 ± 7.81 a	29.44 ± 4.71 bc	10.17 ± 3.30 abc	31.00 ± 8.13 ab
N100	58.44 ± 10.88 a	29.39 ± 3.97 bc	8.72 ± 2.82 c	30.89 ± 7.58 ab
N150	59.42 ± 10.31 a	28.83 ± 3.90 cd	10.50 ± 4.01 abc	34.89 ± 17.66 a
S50	57.58 ± 9.57 a	30.22 ± 3.84 abc	13.61 ± 3.82 a	28.33 ± 6.11 bc
S100	58.00 ± 6.78 a	29.50 ± 3.73 bc	9.11 ± 2.90 bc	28.44 ± 6.16 abc
S150	58.61 ± 8.73 a	31.11 ± 3.27 abc	11.28 ± 4.01 abc	29.67 ± 8.93 ab
U50	59.50 ± 8.16 a	31.06 ± 3.32 abc	13.11 ± 3.51 ab	28.22 ± 4.05 bc
U100	56.50 ± 6.72 a	32.39 ± 3.33 a	13.06 ± 4.53 ab	28.78 ± 6.44 abc
U150	56.63 ± 9.20 a	32.00 ± 3.80 ab	11.67 ± 3.92 abc	29.17 ± 7.39 ab
**Total average**	**56.63 ± 9.20**	**30.06 ± 4.06**	**10.97 ± 3.76**	**29.21 ± 8.80**

^1^ Values are expressed as the mean (n = 18) ± standard deviation. Mean values with different letters (a, b, c, d) within the same column are significantly different (α = 0.05) according to Duncan’s test. Abbreviations are as follows: C—stevia plants grown without the addition of nitrogen fertiliser; N50, N100, N150—stevia plants grown with the addition of 50/100/150 kg of N (in the form of ammonium nitrate) per ha of the field; S50, S100, S150—stevia plants grown with the addition of 50/100/150 kg of N (in the form of ammonium sulfate) per ha of the field; U50, U100, U150—stevia plants grown with the addition of 50/100/150 kg of N (in the form of urea) per ha of the field.

**Table 5:** Dry mass in the stevia leaves of stevia plants grown with different nitrogen fertilisation treatments.

Sample ^1^	Dry Mass Content in Stevia Leaves [%]	
	July	September
C	24.47 ± 4.06 a	28.36 ± 3.47 a
N50	23.59 ± 2.42 a	29.74 ± 3.90 a
N100	26.10 ± 5.21 a	28.98 ± 1.84 a
N150	23.52 ± 1.42 a	27.44 ± 3.26 a
S50	23.80 ± 3.78 a	29.76 ± 2.21 a
S100	23.37 ± 2.93 a	28.64 ± 2.35 a
S150	23.62 ± 3.00 a	28.24 ± 2.18 a
U50	24.41 ± 2.25 a	30.04 ± 5.06 a
U100	22.97 ± 3.50 a	28.59 ± 5.46 a
U150	23.99 ± 1.99 a	28.05 ± 2.79 a
**Total average**	**23.98 ± 3.18**	**28.78 ± 3.72**

^1^ Values are expressed as the mean (n = 9) ± standard deviation. Mean values with letters (a) within the same column are significantly different (α = 0.05) according to Duncan’s test. Abbreviations are as follows: C—stevia plants grown without the addition of nitrogen fertiliser; N50, N100, N150—stevia plants grown with the addition of 50/100/150 kg of N (in the form of ammonium nitrate) per ha of the field; S50, S100, S150—stevia plants grown with the addition of 50/100/150 kg of N (in the form of ammonium sulfate) per ha of the field; U50, U100, U150—stevia plants grown with the addition of 50/100/150 kg of N (in the form of urea) per ha of the field.

**Table 6:** Element concentration in stevia leaves of stevia plants grown with different nitrogen fertilisation treatments.

Sample ^1^	Calcium		Magnesium		Phosphorus		Potassium	
	[mg·100 g^−1^ of d.m.]							
	July	September	July	September	July	September	July	September
C	770.89 ± 49.90 d	993.67 ± 96.77 c	168.89 ± 13.42 ab	229.44 ± 14.26 b	119.60 ± 14.30 b	177.10 ± 18.18 bc	2490.33 ± 205.89 b	2938.89 ± 248.01
N50	794.44 ± 96.65 d	969.44 ± 89.39 bc	159.44 ± 17.66 b	218.89 ± 26.49 b	143.50 ± 11.54 a	173.49 ± 21.45 c	2802.78 ± 112.11 a	3018.11 ± 258.45 ab
N100	816.67 ± 108.97 d	1069.44 ± 108.89 ab	153.33 ± 10.52 b	248.33 ± 26.93 a	146.28 ± 11.25 a	153.08 ± 17.45 d	2468.11 ± 224.53 b	3034.78 ± 257.80 ab
N150	890.33 ± 43.12 bcd	1188.44 ± 125.18 a	156.11 ± 15.60 b	218.89 ± 27.81 b	108.21 ± 6.45 bc	140.58 ± 14.25 de	2616.67 ± 307.97 ab	2797.22 ± 138.66 ab
S50	836.11 ± 45.26 d	994.44 ± 116.91 b	173.33 ± 20.87 a	259.44 ± 27.91 a	140.44 ± 13.60 a	180.86 ± 10.56 b	2521.00 ± 225.03 b	3216.78 ± 173.46 a
S100	861.11 ± 33.33 cd	1098.11 ± 141.23 ab	168.89 ± 27.36 ab	246.67 ± 18.24 ab	131.53 ± 11.92 ab	201.01 ± 9.92 b	2457.00 ± 270.82 b	3050.00 ± 260.49 ab
S150	991.67 ± 46.77 b	1162.00 ± 149.53 a	175.56 ± 14.46 a	240.00 ± 16.34 ab	100.72 ± 9.49 c	148.79 ± 12.05 d	2275.00 ± 222.42 c	2841.78 ± 100.66 ab
U50	887.56 ± 79.45 bcd	863.89 ± 58.03 c	183.33 ± 27.36 a	214.44 ± 25.65 a	128.09 ± 9.05 ab	218.77 ± 8.28 a	2497.33 ± 262.11 b	3048.67 ± 182.52 ab
U100	976.44 ± 111.97 bc	1076.44 ± 132.36 ab	160.56 ± 18.45 ab	222.22 ± 18.15 b	116.69 ± 7.05 b	158.63 ± 9.66 d	2534.78 ± 288.41 ab	3183.33 ± 178.66 a
U150	1144.44 ± 298.64 a	1119.44 ± 110.64 a	152.78 ± 24.18 b	227.22 ± 20.73 b	104.62 ± 8.48 bc	131.13 ± 9.42 e	2298.67 ± 320.36 bc	2534.78 ± 180.53 b
**Total average**	**896.97 ± 105.14**	**1053.53 ± 146.57**	**165.22 ± 20.94**	**232.56 ± 28.10**	**123.97 ± 11.24**	**168.34 ± 14.04**	**2496.17 ± 273.53**	**2966.43 ± 206.73**

^1^ Values are expressed as the mean (n = 9) ± standard deviation. Mean values with different letters (a, b, c, d, e) within the same column are significantly different (α = 0.05) according to Duncan’s test. Abbreviations are as follows: C—stevia plants grown without the addition of nitrogen fertiliser; N50, N100, N150—stevia plants grown with the addition of 50/100/150 kg of N (in the form of ammonium nitrate) per ha of the field; S50, S100, S150—stevia plants grown with the addition of 50/100/150 kg of N (in the form of ammonium sulfate) per ha of the field; U50, U100, U150—stevia plants grown with the addition of 50/100/150 kg of N (in the form of urea) per ha of the field.

**Table 7:** Concentration of ascorbic acids, phenolic compounds, reducing sugars and nitrate ions in stevia leaves of stevia plants grown with different nitrogen fertilisation treatments.

Sample ^1^	Ascorbic Acid		Phenolic Compounds		Reducing Sugars		Nitrate Ions (NO_3_^−^)	
	[mg·100 g^−1^ of f.m.]						[mg·kg^−1^ of d.m.]	
	July	September	July	September	July	September	July	September
C	110.12 ± 5.24 c	135.42 ± 16.22 b	262.21 ± 23.35 b	170.52 ± 18.04 d	1.07 ± 0.14 a	2.30 ± 0.29 a	1501.11 ± 393.81 b	890.33 ± 263.28 a
N50	128.24 ± 26.77 ab	148.13 ± 10.65 ab	249.66 ± 28.44 b	251.72 ± 25.54 c	0.89 ± 0.11 ab	2.41 ± 0.28 a	1657.44 ± 331.26 b	952.44 ± 381.94 a
N100	114.14 ± 6.05 bc	142.81 ± 11.30 ab	286.57 ± 28.94 ab	295.56 ± 26.15 b	0.94 ± 0.25 ab	2.31 ± 0.24 a	2116.78 ± 431.57 ab	1246.56 ± 374.77 a
N150	109.27 ± 6.90 c	135.89 ± 10.53 b	326.30 ± 30.70 a	348.58 ± 28.02 a	0.91 ± 0.11 ab	1.99 ± 0.27 a	2004.67 ± 423.43 ab	1225.67 ± 357.26 a
S50	124.5 ± 8.50 abc	137.94 ± 10.11 ab	203.07 ± 37.02 c	251.57 ± 22.63 c	1.04 ± 0.15 a	2.09 ± 0.21 a	1552.11 ± 341.55 b	962.11 ± 463.64 a
S100	118.48 ± 13.28 bc	142.67 ± 8.36 ab	231.93 ± 31.97 bc	272.43 ± 23.44 bc	0.94 ± 0.09 ab	2.08 ± 0.25 a	1911.44 ± 207.36 ab	1027.33 ± 389.75 a
S150	111.12 ± 6.45 c	149.28 ± 7.95 a	309.37 ± 35.52 a	293.96 ± 18.92 b	0.84 ± 0.21 b	2.03 ± 0.37 a	1764.67 ± 511.72 ab	1372.44 ± 406.16 a
U50	135.54 ± 27.56 a	138.57 ± 23.64 ab	236.21 ± 43.63 bc	304.51 ± 37.92 ab	0.98 ± 0.24 ab	1.86 ± 0.49 a	1621.67 ± 449.14 b	881.44 ± 348.14 a
U100	120.50 ± 12.44 bc	140.54 ± 6.45 ab	346.07 ± 37.43 a	328.77 ± 38.36 a	0.92 ± 0.19 ab	2.02 ± 0.37 a	2329.44 ± 300.93 a	1045.22 ± 436.89 a
U150	112.94 ± 5.46 bc	137.04 ± 6.82 ab	302.31 ± 40.81 a	172.43 ± 21.44 d	0.99 ± 0.06 ab	2.07 ± 0.39 a	1765.67 ± 433.04 ab	1295.78 ± 427.80 a
**Total average**	**118.49 ± 16.20**	**140.83 ± 12.36**	**275.37 ± 38.16**	**269.00 ± 46.08**	**0.95 ± 0.17**	**2.12 ± 0.69**	**1822.50 ± 343.74**	**1089.93 ± 348.06**

^1^ Values are expressed as the mean (n = 9) ± standard deviation. Mean values with different letters (a, b, c, d) within the same column are significantly different (α = 0.05) according to Duncan’s test. Abbreviations are as follows: C—stevia plants grown without the addition of nitrogen fertiliser; N50, N100, N150—stevia plants grown with the addition of 50/100/150 kg of N (in the form of ammonium nitrate) per ha of the field; S50, S100, S150—stevia plants grown with the addition of 50/100/150 kg of N (in the form of ammonium sulfate) per ha of the field; U50, U100, U150—stevia plants grown with the addition of 50/100/150 kg of N (in the form of urea) per ha of the field.

**Table 8:** Concentration of chlorophylls and carotenoids in stevia leaves of stevia plants grown with different nitrogen fertilisation treatments.

Sample ^1^	Chlorophyll A		Chlorophyll B		Chlorophyll A + B		Carotenoid	
	[mg·g^−1^ of f.m.]							
	July	September	July	September	July	September	July	September
C	0.87 ± 0.25 ab	0.77 ± 0.14 ab	0.37 ± 0.13 a	0.36 ± 0.02 bc	1.23 ± 0.35 ab	1.13 ± 0.12 ab	2.52 ± 0.59 a	2.26 ± 0.33 a
N50	0.66 ± 0.13 b	0.66 ± 0.11 b	0.31 ± 0.15 a	0.37 ± 0.11 bc	0.98 ± 0.27 b	1.03 ± 0.21 ab	2.16 ± 0.39 a	2.07 ± 0.54 a
N100	0.82 ± 0.18 ab	0.80 ± 0.25 a	0.35 ± 0.18 a	0.51 ± 0.19 a	1.17 ± 0.34 ab	1.31 ± 0.39 a	2.55 ± 0.32 a	2.58 ± 0.70 a
N150	0.76 ± 0.15 ab	0.84 ± 0.22 a	0.35 ± 0.13 a	0.43 ± 0.14 ab	1.11 ± 0.27 ab	1.27 ± 0.34 ab	2.34 ± 0.50 a	2.64 ± 0.74 a
S50	0.75 ± 0.17 ab	0.78 ± 0.13 a	0.33 ± 0.15 a	0.45 ± 0.12 ab	1.08 ± 0.32 ab	1.22 ± 0.20 ab	2.36 ± 0.56 a	2.50 ± 0.44 a
S100	0.84 ± 0.21 ab	0.71 ± 0.27 ab	0.39 ± 0.15 a	0.28 ± 0.15 c	1.23 ± 0.34 ab	0.99 ± 0.40 b	2.44 ± 0.58 a	2.25 ± 0.77 a
S150	0.91 ± 0.31 ab	0.74 ± 0.20 ab	0.41 ± 0.10 a	0.45 ± 0.16 ab	1.32 ± 0.38 ab	1.19 ± 0.31 ab	2.68 ± 0.74 a	2.46 ± 0.58 a
U50	0.98 ± 0.19 a	0.75 ± 0.15 ab	0.43 ± 0.15 a	0.40 ± 0.11 abc	1.41 ± 0.31 a	1.15 ± 0.25 ab	2.60 ± 0.45 a	2.24 ± 0.49 a
U100	0.90 ± 0.26 ab	0.86 ± 0.11 a	0.38 ± 0.17 a	0.46 ± 0.07 ab	1.28 ± 0.40 ab	1.32 ± 0.10 a	2.53 ± 0.69 a	2.65 ± 0.30 a
U150	0.89 ± 0.30 ab	0.83 ± 0.21 a	0.42 ± 0.14 a	0.44 ± 0.12 ab	1.31 ± 0.38 ab	1.27 ± 0.30 ab	2.51 ± 0.75 a	2.53 ± 0.48 a
**Total average**	**0.84 ± 0.23**	**0.77 ± 0.19**	**0.38 ± 0.14**	**0.41 ± 0.14**	**1.21 ± 0.34**	**1.19 ± 0.29**	**2.47 ± 0.56**	**2.42 ± 0.56**

^1^ Values are expressed as the mean (n = 9) ± standard deviation. Mean values with different letters (a, b, c) within the same column are significantly different (α = 0.05) according to Duncan’s test. Abbreviations are as follows: C—stevia plants grown without the addition of nitrogen fertiliser; N50, N100, N150—stevia plants grown with the addition of 50/100/150 kg of N (in the form of ammonium nitrate) per ha of the field; S50, S100, S150—stevia plants grown with the addition of 50/100/150 kg of N (in the form of ammonium sulfate) per ha of the field; U50, U100, U150—stevia plants grown with the addition of 50/100/150 kg of N (in the form of urea) per ha of the field.

**Table 9:** Concentration of steviosides in stevia leaves of stevia plants grown with different nitrogen fertilisation treatments.

Sample ^1^	Stevioside	Rebaudoside A
	[mg·100 g^−1^ of d.m.]	
C	135.14 ± 6.33 fg	352.85 ± 6.33 g
N50	140.50 ± 5.32 e	785.20 ± 8.96 ef
N100	156.6 ± 1.12 d	1093.08 ± 106.85 cd
N150	134.11 ± 1.65 fg	1229.89 ± 18.42 c
S50	111.69 ± 1.53 h	602.18 ± 82.60 fg
S100	214.26 ± 11.45 b	1493.53 ± 133.78 b
S150	262.36 ± 17.13 a	2186.62 ± 390.54 a
U50	108.91 ± 0.70 h	817.05 ± 54.46 ef
U100	122.53 ± 6.54 gh	935.04 ± 79.86 de
U150	182.46 ± 8.59 c	490.24 ± 27.96 g
**Total average**	**156.86 ± 17.99**	**998.57 ± 234.99**

^1^ Values are expressed as the mean (n = 9) ± standard deviation. Mean values with different letters (a, b, c, d, e, f, g, h) within the same column are significantly different (α = 0.05) according to Duncan’s test. Abbreviations are as follows: C—stevia plants grown without the addition of nitrogen fertiliser; N50, N100, N150—stevia plants grown with the addition of 50/100/150 kg of N (in the form of ammonium nitrate) per ha of the field; S50, S100, S150—stevia plants grown with the addition of 50/100/150 kg of N (in the form of ammonium sulfate) per ha of the field; U50, U100, U150—stevia plants grown with the addition of 50/100/150 kg of N (in the form of urea) per ha of the field.

**Table 10:** Temperature in the area of the stevia plantation during the time of the experiment and the average temperature in the previous years.

Month	Decade of the Month	Year 2014	Year 2015	Year 2016	Average Temperature in the Month during the Years 1981–2010
		[°C]			
May	1st 10-day period	11.4	13.3	15.4	14.2
	2nd 10-day period	12.2	13.0	15.5	
	3rd 10-day period	17.3	14.0	20.9	
	Monthly average	13.8	13.4	17.3	
June	1st 10-day period	18.4	18.1	21.1	17.0
	2nd 10-day period	16.6	16.4	18.5	
	3rd 10-day period	15.7	15.8	23.8	
	Monthly average	16.9	16.8	21.1	
July	1st 10-day period	20.5	20.5	20.5	19.2
	2nd 10-day period	22.5	20.3	20.1	
	3rd 10-day period	22.6	19.9	23.8	
	Monthly average	21.9	20.2	21.5	
August	1st 10-day period	21.7	27.2	20.4	18.5
	2nd 10-day period	17.4	25.2	19.7	
	3rd 10-day period	15.9	23.1	21.3	
	Monthly average	18.3	25.2	20.5	
September	1st 10-day period	17.2	17.1	21.2	13.9
	2nd 10-day period	18.6	18.8	19.3	
	3rd 10-day period	14.0	14.1	15.0	
	Monthly average	16.6	16.7	18.5	

**Table 11:** Level of rainfall in the area of stevia plantation during the time of the experiment and the average level of rainfall in the previous years.

Month	10-Day Period of the Month	Year 2014	Year 2015	Year 2016	Average Rainfall in the Month during the Years 1981–2010
		[mm of Rainfall]			
May	1st 10-day period	30.3	12.3	14.0	57.0
	2nd 10-day period	33.6	14.0	12.0	
	3rd 10-day period	42.8	0.5	0.0	
	Monthly average	106.7	26.8	26.0	
June	1st 10-day period	8.2	11.0	9.4	69.9
	2nd 10-day period	0.3	23.1	59.0	
	3rd 10-day period	15.4	32.2	0.8	
	Monthly average	23.9	66.3	69.2	
July	1st 10-day period	20.0	11.0	39.5	83.4
	2nd 10-day period	0.0	38.9	70.5	
	3rd 10-day period	26.1	3.3	24.5	
	Monthly average	46.1	53.2	134.5	
August	1st 10-day period	17.5	0.0	5.8	71.0
	2nd 10-day period	13.1	0.0	5.0	
	3rd 10-day period	35.0	2.1	13.3	
	Monthly average	65.6	2.1	24.1	
September	1st 10-day period	24.1	16.4	11.5	45.2
	2nd 10-day period	21.6	0.0	45.8	
	3rd 10-day period	14.3	0.0	0.0	
	Monthly average	60.0	16.4	57.3	

## Data Availability

The data presented in this study are available on request from the corresponding author.
